# Contribution of Aldehydes and Their Derivatives to Antimicrobial and Immunomodulatory Activities

**DOI:** 10.3390/molecules27113589

**Published:** 2022-06-02

**Authors:** Mariam Nasser Aljaafari, Maryam Abdulraheem Alkhoori, Mohammed Hag-Ali, Wan-Hee Cheng, Swee-Hua-Erin Lim, Jiun-Yan Loh, Kok-Song Lai

**Affiliations:** 1 Health Sciences Division, Abu Dhabi Women’s College, Higher Colleges of Technology, Abu Dhabi 41012, United Arab Emirates; h00349760@hct.ac.ae (M.N.A.); h00391358@hct.ac.ae (M.A.A.); lerin@hct.ac.ae (S.-H.-E.L.); 2 Higher Colleges of Technology Headquarters, CERT Building, Abu Dhabi 41012, United Arab Emirates; hagali@hct.ac.ae; 3 Faculty of Health and Life Sciences, INTI International University, Persiaran Perdana BBN, Putra Nilai, Nilai 71800, Malaysia; wanhee.cheng@newinti.edu.my; 4 Centre of Research for Advanced Aquaculture (CORAA), UCSI University, Kuala Lumpur 56000, Malaysia

**Keywords:** aldehydes, derivatives, essential oils, immunomodulatory activity, antimicrobial activity

## Abstract

Essential oils (EOs) are intricate combinations of evaporative compounds produced by aromatic plants and extracted by distillation or expression. EOs are natural secondary metabolites derived from plants and have been found to be useful in food and nutraceutical manufacturing, perfumery and cosmetics; they have also been found to alleviate the phenomenon of antimicrobial resistance (AMR) in addition to functioning as antibacterial and antifungal agents, balancing menstrual cycles and being efficacious as an immune system booster. Several main aldehyde constituents can be found in different types of EOs, and thus, aldehydes and their derivatives will be the main focus of this study with regard to their antimicrobial, antioxidative, anti-inflammatory and immunomodulatory effects. This brief study also explores the activity of aldehydes and their derivatives against pathogenic bacteria for future use in the clinical setting.

## 1. Introduction

**Figure 1:**
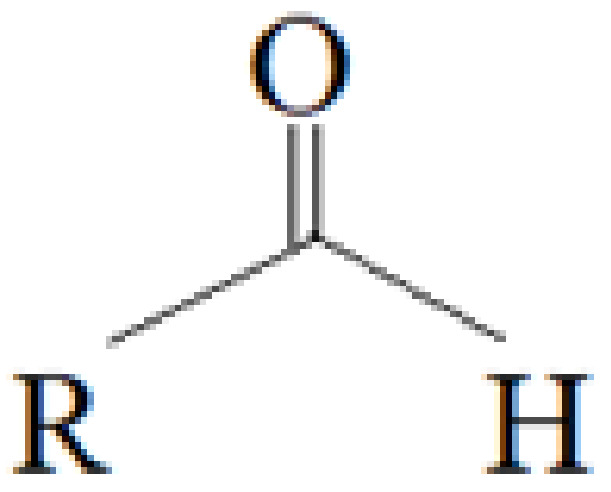
Aldehyde’s chemical structure.

Aldehydes are a class of highly reactive and toxic chemicals with several uses in the industrial sector. Aldehydes are derived from alcohols after the removal of hydrogen in a process called dehydrogenation [1]. The structure of aldehyde is shown in Figure 1 below [2].

Aldehydes are also known to affect both olfaction and gestation senses in mammals. Aldehyde odorants such as hexanal and cinnamaldehyde bind to the receptors bonded to the G-protein, which triggers reaction cascades and causes the perception of mammalian senses [3]. The simplest aldehyde is formaldehyde, in which the carbonyl group binds to two hydrogens. Different types of aldehydes include fatty aldehydes, such as hexanal, decanal, and octanal, with fresh apple, orange peel, and citrus odors, respectively, whereas aromatic aldehydes include cinnamaldehyde and benzaldehyde, with cinnamon and almond odors, respectively. Moreover, notable terpenoid aldehydes are represented by safranal and citral, which gives saffron and lemon their aroma [4,5]. See Table 1 for the different types of aldeydes.

Aromatic aldehydes also give plants their fragrances and flavors; for example, benzaldehyde is an aldehyde that can be used in the perfumery, pharmaceuticals, and flavoring industries [6,7]. Moreover, anisaldehyde, a compound consisting of a benzene ring with formyl and methoxy groups, is responsible for the natural sweet blossom fragrance, while vanillin extracted from the vanilla bean is responsible for the vanilla fragrance. Other than being extracted from plants or being chemically synthesized, aldehydes can also be produced in most microorganisms. This pathway can be helpful as it has been shown to minimize the rapid conversion of aldehydes into alcohols if needed to produce some challenging biochemical classes, as recent studies that used *Escherichia coli* have proven; this pathway helps to broaden the usage of aldehydes as intermediates [4,8]. Aldehydes may be found in small quantities in essential oils (EOs) within plants. EOs are a volatile, concentrated mixture of organic compounds that are produced mainly as a defense mechanism in plants [9,10,11]. In addition to their physiological roles as pheromones and phytohormones, EO compounds provide some advantages that have been discovered and potentially hold more applications yet to be known. One of the crucial advantages of the extracted EOs from plants is their possible application as a replacement for potential antimicrobial agents to minimize the proliferation of foodborne pathogenic microorganisms for food preservation [9,10,12,13]. Moreover, EOs have been shown to cure infectious skin diseases such as burns, ulcers, deep wounds and acne that are caused by *Cutibacterium acnes* and folliculitis caused by *Staphylococcus aureus* and *Pseudomonas aeruginosa* [12]. To date, approximately 90 types of essential oils have been reported to be useful for dermatological recuperation purposes [13].

EOs are composed of different compounds of monoterpenes, sesquiterpenes, and phenylpropanoids, such as alcohols, aldehydes, carbohydrates and ketones [14,15,16]. Although they are present in small amounts in EOs, aldehydes are considered key contributors to an EO’s overall odor due to their potent fragrances [17]. For example, the chemical composition of the *Melissa officinalis* L. EO, which is from the family *Lamiaceae*, was determined and then analyzed by GC/MS and GC-FID analysis. It showed that only 6.30% citronellal is present in the oil, and although it is considered a minor constituent, it is one of the main contributors to the oil’s fragrance [18,19]. Aldehydes need to be used appropriately as they are not only respiratory irritants after inhalation but also dermal irritants when used excessively on the skin topically [20]. Examples of aldehydes that can be found in EOs are cinnamaldehyde and benzaldehyde (*Cinnamomum cassia* EO), citronellal, geranial and neral (*Cymbopogon citratus* EO), citral (Germany *M. officinalis* EO), and perillaldehyde (*Perilla frutescens* EO) [16,21,22,23,24]. Aldehydes are also used to synthesize chemical compounds, which makes them important in the field of organic chemistry.

**Figure 2:**
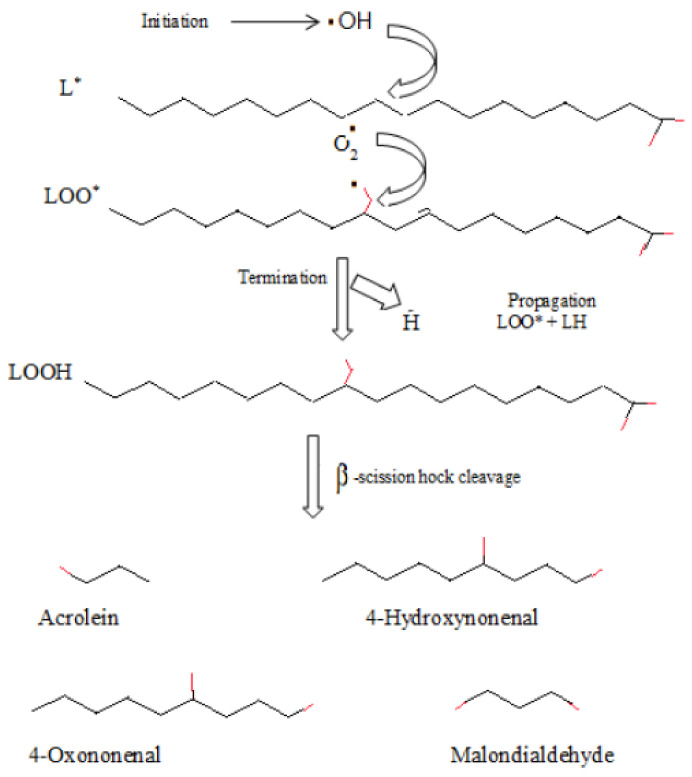
The three steps of the lipid peroxidation process, where L* represents lipid radical.

Aldehydes can be generated by oxidation of the primary alcohols, dehydrogenation of alcohols, via oxidation of methylbenzene or derived from hydrocarbons and the reduction of esters. The first process is hard to perform as the reagent that is used to oxidize alcohols can cause aldehydes to be oxidized as well [25,26]. Reactive aldehydes can be produced by both enzymatic and non-enzymatic mechanisms. For example, the nonenzymatic free radical mechanism is one of the important ways to produce a variety of aldehydes, while lipid peroxidation, which is a simple oxidative lipid degradation reaction, can produce both aldehydes and hydroperoxides [26,27]. Lipid peroxidation occurs by initiation, which happens by the bis-allylic hydrogen removal from a lipid chain to cause a lipid radical (L˙). Each initiation chain will result in 200 to 400 propagation cycles [28]. Propagation will occur by the addition of oxygen to the radical with carbon in the center, and the L˙ will be converted quickly to an oxygen-centered peroxyl radical. Then, this radical will react with another lipid chain to produce L˙ and an unstable lipid hydroperoxide, giving rise to peroxyl and alkoxyl radicals. After propagation, termination of the reaction will occur if two free radical species combine, giving rise to a non-radical species [29,30]. See Figure 2 for the three steps of the lipid peroxidation process. This review will focus on aldehydes that are found in EO and their derivatives, as well as their mechanisms of action in antimicrobial and immunomodulatory activities.

## 2. Aldehydes from EOs and Their Derivatives

Aldehydes are large organic compounds that consist of a single carbon atom, a double bond with an oxygen atom, a single bond with a hydrogen atom and a single bond with the side chain R group (—CHO). There are several ways to synthesize aldehydes, such as the oxidation process to form aldehydes and the hydration process of an alkyne to form aldehydes [31]. Aldehydes can be derived from several types of EO with high concentrations, such as Cassia oil; its main aldehyde constituent is benzaldehyde. Additionally, cinnamon EO’s main aldehyde constituent is cinnamaldehyde; lemongrass and *Melissa* EO’s main aldehyde constituent is geranial, and cilantro EO’s is decenal. In this regard, the most popularly used aldehyde constituents in EOs are cinnamaldehyde and geranial [32].

Essential oils can be classified into two categories based on chemical compounds; the hydrocarbons and oxygenated compound aldehydes are retained as one of the oxygenated compounds within EOs. For example, one of the aldehyde derivatives in cinnamon EO is trans-cinnamaldehyde (TCA). Cinnamon powder has been proven to have valuable impacts on stroke patients as a stroke therapy. Post-ischemic irritation incites neuronal cell harm after stroke, and enactment of microglia, specifically, has been thought of as the fundamental reason for proinflammatory and neurotoxic variables [33].

*Cinnamomum cassia* has numerous practical properties. For example, it has been used as an antimicrobial, in addition to being an agent in controlling high blood pressure, as well as for kidney disorders and cancer [34].

Lemongrass essential oil, extracted from *Cymbopogon citratus*, which is the lemongrass plant, has been utilized since antiquated occasions in people for medication as a solution to improve blood flow, balance menstrual cycles, and advance assimilation or increment invulnerability. It is additionally used in fragrances, cleansers, and drugs. The highest rate of chemical composition that has been found in the lemongrass essential oil is citral, which comprises neral, an acyclic monoterpene aldehyde, and geraniol [35,36]. Recently, lemongrass essential oil was shown to inhibit bacterial and fungal growth [37].

Cilantro oil is another EO that contains aldehyde. The coriander plant extraction method was conducted after drying the plant itself under the sun and in microwave and freeze-drying processes. On the therapeutical side, coriander EO has presented a significant impact on patients with Alzheimer’s disease, such as improving the patient’s memory, apart from controlling the cholesterol levels and managing anticholinesterase activity. Furthermore, coriander EO has also shown antibacterial and antifungal spread [38].

*Melissa officinalis* essential oil (MOEO) has demonstrated several advantages after oral administration to Wistar rats in an exploratory model of diabetic hyperalgesia, indicating that MOEO may have potential as a treatment for excruciating diabetic neuropathy [39]. MOEO has been used against viral infections, and the outcomes showed it has an impact on the infections. More specifically, it has shown inhibitory effects against the avian influenza virus (AIV). The results of the investigation showed that MOEO could repress the flu virus infection replication process through various replication cycle steps, specifically when in contact with virus particles [40]. See Table 2 for EOs containing aldehydes, their application and advantages.

## 3. Antimicrobial Activity of Aldehydes

Aldehydes have been shown to contribute to antimicrobial activity. The process starts with mechanical destruction from the outer bacterial cell membrane. In addition, EOs have the ability to dissolve in lipids, which supports the fact that they are considered lipophilic. EOs aim to convert the rigid phospholipid layers, fatty acids and polysaccharides to flexible and porous layers, which aids in softening the membrane [44,45].

Cinnamaldehyde is one of the important aldehyde categories since it has a great role in antimicrobial activity [46]. The phase inversion temperature method was conducted by Rao and McClements in 2010 in order to perform the oil extraction procedure, which is basically based on heating the oil/water mixture and then cooling the mixture by stirring [47]. The effect of oil phase composition on the minimum inhibitory concentration (MIC) of cinnamon oil nanoemulsions against four foodborne pathogens, *E. coli*, *Salmonella typhimurium*, *Staphylococcus aureus*, and *Vibrio parahaemolyticus*, was calculated by the phase inversion temperature method. As a result, the cinnamon oil nanoemulsion generated from this condition maintained a good consistency and antibacterial action over time. Although the MICs of cinnamon oil nanoemulsion were greater than those of bulk cinnamon oil, preliminary in vitro time-kill tests revealed that cinnamon oil nanoemulsion exhibited a quick and sustained bacteriostatic impact after being in contact with four foodborne pathogens: *E. coli*, *Salmonella typhimurium*, *Staphylococcus aureus*, and *Vibrio parahaemolyticus* [42]. Cinnamaldehyde was shown to inhibit the growth of the four tested pathogens within one day of incubation [46,48]. Furthermore, a broth volatilization chequerboard method was conducted on *Cinnamomum cassia* mixed with 8-hydroxyquinoline versus *Staphylococcus aureus* varieties in steam and liquid forms. The results showed that *Staphylococcus aureus* was inhibited when treated with *Cinnamomum cassia* essential oil with 8-hydroxyquinoline during the extraction process [49]. Cinnamaldehyde also works effectively against the inhibition of bacterial biofilm formation [50].

Lemongrass essential oil has revealed very significant antimicrobial action against several types of bacteria, molds and yeasts [51]. A test was done on lemongrass essential oil that was exposed to two types of fungal pathogens. *Ascosphaera apis* and *Pseudogymnoascus destructants* showed that both compounds inhibited the growth of the fungi, potentially having possible antifungal activity of other fungal types as well [52]. *Coriandrum sativum* L., which is known as the coriander plant, has been tested on Gram-negative and Gram-positive bacteria and pathogenic skin fungus. This shows the potential impact of coriander oil on antimicrobial activity [53]. The antimicrobial activity of *M. officinalis* essential oil tested using disc diffusion agar showed that *M. officinalis* has a high specificity of antimicrobial effect on *Streptococcus pyogenes* at 8 mg/mL, at 15.10 ± 0.52, compared to *S. epidermidis*, which was 14.50 ± 0.50 [54].

## 4. Immuno-Modulatory Activities of Aldehydes

Aldehydes are a chemical class with several uses in the industrial sector. However, the toxicity of aldehydes makes them practical challenging for extensive use in the commercial and academic sectors [4,8]. Studies on aldehydes have shown different types of activities to be present.

### 4.1. Aldehydes with Immune Effect

#### 4.1.1. Cinnamaldehydes

Cinnamaldehyde is an organic compound found in the cinnamon bark oil, which helps in amino acid decarboxylase inhibition. It also has antibiofilm and anticancer activities [9,55]. Cinnamon EO contains 80% cinnamaldehyde, which is responsible for cinnamon’s natural fragrance [4,56]. Studies have also found that cinnamaldehyde has an antimicrobial, antioxidative, anti-inflammatory and immunomodulatory effect [57]. Cinnamaldehyde blended with thymol has been studied and evaluated in a study for its immune-modulating effect in the poultry immune system by using both a liver cell line and a monocyte/macrophage-like cell line. Results showed that under the testing conditions, the blend of cinnamaldehyde and thymol improved the integrity of the epithelial barrier in poultry liver cells, produced anti-inflammatory cytokines by monocytes and macrophages, and encouraged phagocytic activity, thus increasing in vivo cell membrane integrity and birds’ performance [58]. Another study that evaluated the effect of cinnamaldehyde on cardiac hypertrophy in mice performed aortic banding. After one week, the mice were fed a premixed diet with cinnamaldehyde by catheter. Echocardiography measurements were performed after 8 weeks from the aortic banding, which showed improvement of the abnormal systolic and diastolic pressure by the cinnamaldehyde. Additionally, cardiac fibrosis was decreased [59]. Moreover, studies showed that when the extracellular signal-regulated kinase (ERK) pathway was activated by the pressure overload, cinnamaldehyde could be used to block it [59]. Another study done to evaluate cinnamon, clove, and white thyme on selected bacteria has revealed that cinnamon showed the highest microbial activity, in addition to down-regulating the expression of genes related to apoptosis pathways and the inhibition of interleukin-2 secretion [43].

#### 4.1.2. Geranial

Drugs that are used to treat African and American trypanosomiasis infections cause many side effects; therefore, there is a need to develop alternative drugs [60]. Geranial and neral isolated from ethyl acetate extracts, which are monoterpene aldehydes, were reported to have trypanocidal activity against *Trypanosoma cruzi* with minimum lethal concentrations (MLC) of 3.1 µM [61]. Generally, the monoterpene aldehydes are potent against *T. cruzi* and *Trypanosoma brucei*, but the main challenge is their toxicity to human cells [61]. The immunomodulatory activity of geranial EO and other EO has been evaluated in vivo using rats by studying hemagglutination and hypersensitivity reactions using sheep red blood cells as an antigen and sodium carboxy methyl cellulose as a control. Results showed an increase in hemagglutinating antibody titer and hypersensitivity reaction. In rats immunized with the antigen, cellular immunity has been potentiated; these results show that geranial and other studied EOs can stimulate immune activity by both specific and non-specific mechanisms [62].

Essential oils can be used for their antimicrobial and immunomodulatory effects. However, the exact mechanisms are still unknown and need further research, and the toxicity of some aldehydes due to their high reactivity should be taken into consideration. See Table 3 for a summary of the results of studies on the immunomodulatory activity of aldehydes.

### 4.2. Aldehydes Toxicity

Humans are commonly exposed to aldehydes, and although constant exposure bears health risks, their toxicity mechanisms are still not fully understood. This could be because of the diversity of structures, chemical reactions, and targets. In a short mechanistic view, a study analysis describing both endogenous and environmental aldehydes by their electrophilicity and relative softness has shown that soft unsaturated aldehydes and the soft nucleophilic thiolate sites react together on the same cysteine residues in enzymes. In contrast, hard alkanals go with hard nucleophiles, and these specific reactions show that it mediates toxicity by weakening the macromolecule functions that are important in cytophysiological activity. However, there is a need for a better understanding of how these specific reactions affect the targets of macromolecules [63].

#### 4.2.1. Acetaldehyde

Acetaldehyde is ethanol’s first metabolite with a formula (C_2_H_4_O) that can be formed naturally in the body and plants and found in fruits, milk, and cheese [64]. A study done on rat gastric epithelial cells to elucidate the effect of acetaldehyde by the measurement of the electron paramagnetic resonance showed that acetaldehyde induces oxidative stress and acts as gastric epithelial cell’s necrotizing factor [65]. As a result of oxidative stress, when cells are exposed to 0.05–0.5% acetaldehyde, lipid peroxidation is induced. Moreover, the cell viability test result shows that cell viability after the addition of acetaldehyde into the culture medium with a concentration of 0.01% showed a cytotoxic effect 30 min after exposure. After 60 min exposure to acetaldehyde, cells demonstrated more than 0.5% necrosis cell death, which proves that acetaldehyde can induce cell death [65].

#### 4.2.2. Formaldehyde

Formaldehyde is a colorless organic compound with the (CH₂O) formula [66]. Updated studies from the past 8 years that primarily evaluated the in vivo and in vitro information about formaldehyde toxicity and its cytotoxicity on humans, mice, and rats have shown that human health was significantly harmed after the occupational exposure to formaldehyde. For example, the lungs, bone marrow, cells and brain were affected due to formaldehyde exposure [67]. Moreover, formaldehyde was classified as an environmental contaminant and a carcinogen according to the International Agency for Research on Cancer in 2004, but it was inconclusive due to the limitations in designing the population studies [68,69]. Furthermore, many retrospective studies on humans have provided evidence for the association between developmental effects and maternal exposure. They found that pregnant women exposed to formaldehyde had increased abortion risks, coupled with adverse pregnancy outcomes. Studies on animals proved that the association between the developmental and reproductive effect of formaldehydes exists regardless of dose, and such exposure leads to genotoxicity, alteration of enzymatic function, hormones, apoptosis, oxidative stress, and toxicogenomic repercussions [69]. Both molecular epidemiologic and mechanistic and animal studies will help further understand the toxicity of formaldehyde on biological systems.

#### 4.2.3. Benzaldehyde

Benzaldehyde is an aromatic aldehyde commonly used in cosmetics, food additives and fragrances [70]. In general, it is considered safe to be used in the food industry. Studies have reported very little acute toxicity or no adverse effect of using benzaldehyde. For instance, the oral LD50 of benzaldehyde in mice and rats was reported to be in the range of 800 to 250 mg/kg [70]. Meanwhile, short-term oral studies in mice and rats recorded no observed adverse effect levels [71]. However, repeated inhalation of volatilized benzaldehyde caused ocular and nasal irritation in rabbits at 500 ppm [72]. In addition, a drop of undiluted benzaldehyde was reported to induce irritation in rabbit eyes, leading to edema, erythema and pain [73].

#### 4.2.4. Cinnamaldehyde

Cinnamaldehyde, with the formula of C_6_H_5_CH=CHCHO, is an organic compound commonly utilized in the flavorant and fragrance industries [74]. It is a natural active ingredient that is well-tolerated in humans and animals [75]. The Food and Drug Administration (FDA) and the council of Europe have recognized cinnamaldehyde as safe with a recommended daily intake of 1.25 mg/kg [76]. Cinnamaldehyde has also been reported to possess various health benefits. For instance, it is used in traditional Chinese medicine for gastritis, indigestion, blood circulation disorders, and inflammation [77,78]. In addition, cinnamaldehyde could remove natural or chemical toxicities such as ochratoxin A and protect human health [79,80].

## 5. Future Prospect

Aldehydes are highly reactive chemical classes that have been the interest of academia and industry. Due to the diverse applications of aldehydes, the aim of a product’s application can help specify the requirement for the aldehyde products. Aldehydes can be used in cosmetics, pharmaceuticals, plastic production, (bio)fuels and perfumery applications [8]. Aldehydes’ high reactivity and toxicity make them difficult to produce; thus, there is a need to develop new technologies to overcome these challenges. The possible solutions for this challenge are complicated due to the differences in toxicity levels and the harmful effect of aldehydes due to several mechanisms acting simultaneously. The development in the understanding of how to slow the reduction of aldehydes to alcohols in living microbes makes it possible to synthesize classes of biochemicals that were hard to synthesize before. Finally, more research is required in order to improve the use of metabolic engineering and biocatalysts with regards to aldehyde production, along with finding solutions to overcome the existing challenges.

## Figures and Tables

**Table 1:** Aldehyde types and their different odors.

Type of Aldehyde	Examples	Odor	References
Fatty aldehydes	Hexanal, decanal, and octanal	Fresh apple, orange peel, and citrus odors	[4,5,6,7]
Aromatic aldehydes	Cinnamaldehyde, anisaldehyde, vanillin and benzaldehyde	Cinnamon, sweet blossom, vanilla and almond odors	[4,5,6,7]
Notable terpenoid aldehydes	Safranal and citral	Saffron and lemon aroma	[4,5,6,7]

**Table 2:** EO, aldehydes, advantages, applications and structures.

EO Name	Source	Main Aldehyde Constituent	Characteristics/Applications	Structure	References
Cassia oil	*Cinnamomum cassia*	Benzaldehyde	- Arthritis alleviation - Immune system booster - Antimicrobial - High blood pressure control	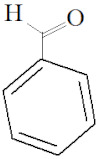	[41]
Cinnamon oil	*C. cassia*	Cinnamaldehyde	- Cholesterol levels reduction - Kidney disorder treatments	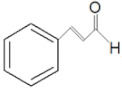	[33,34,42]
Lemongrass oil	*Cymbopogon citratus* leaves	Geranial	- Digestive tract convulsion treatment - Balance menstrual cycles	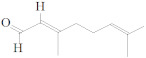	[37]
Melissa oil	*Melissa officinalis*	Geranial	- Diabetes and insomnia treatment - Suppress flu infection replication	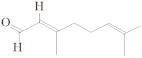	[38,39,43]
Cilantro oil	*Coriandrum sativum* L.	Decenal	- Body detoxification - Antibacterial and antifungal spread	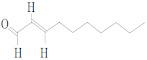	[38]

**Table 3:** Summary of studies’ results for aldehydes’ immunomodulatory activity.

Aldehyde Name	Used	Target	Host	Result	References
Cinnamaldehydes	Cinnamaldehyde + thymol	Immune system	Poultry	Improved epithelial barrier integrity of liver cells Cytokine production by monocytes and macrophages Phagocytic activity Increased in vivo cell membrane integrity and birds performance	[58]
	Cinnamaldehyde	Mice suffering cardiac hypertrophy	Mice	Improved abnormal systolic and diastolic pressure Decreased cardiac fibrosis	[59]
	Cinnamaldehyde	Selected bacteria	Bacteria	High microbial activity Down-regulation of apoptosis genes Inhibition of interleukin-2 secretion	[43]
Geranial	Geranial + neral	Rats *cruzi* Sheep red blood cells (antigen) Sodium carboxy methyl cellulose (control)	Rats	Increased hemagglutinating antibody titer Increased hypersensitivity reaction In immunized rats, potentiated cellular immunity	[62]

## Data Availability

Not applicable.

## References

[1] Speight J.G., Speight J.G. (2017). Chapter 3-Industrial Organic Chemistry. Environmental Organic Chemistry for Engineers.

[2] Tanabe S., Mitsunuma H., Kanai M. (2020). Catalytic Allylation of Aldehydes Using Unactivated Alkenes. J. Am. Chem. Soc..

[3] Li Y., Peterlin Z., Ho J., Yarnitzky T., Liu M.T., Fichman M., Niv M.Y., Matsunami H., Firestein S., Ryan K. (2014). Aldehyde Recognition and Discrimination by Mammalian Odorant Receptors via Functional Group-Specific Hydration Chemistry. ACS Chem. Biol..

[4] Kunjapur A.M., Kristala L.J. (2015). Prather Microbial Engineering for Aldehyde Synthesis. Appl. Environ. Microbiol..

[5] Teranishi R., Wick E.L., Hornstein I., Teranishi R., Wick E.L., Hornstein I. (1999). Flavor Chemistry. Flavor Chemistry: Thirty Years of Progress.

[6] Zhao G., Kuang G., Li J., Hadiatullah H., Chen Z., Wang X., Yao Y., Pan Z.-H., Wang Y. (2020). Characterization of aldehydes and hydroxy acids as the main contribution to the traditional Chinese rose vinegar by flavor and taste analyses. Food Res. Int..

[7] Benzaldehyde. PubChem. https://pubchem.ncbi.nlm.nih.gov/compound/240.

[8] Kazimírová V., Rebroš M. (2021). Production of Aldehydes by Biocatalysis. Int. J. Mol. Sci..

[9] Aljaafari M., Alhosani M.S., Abushelaibi A., Lai K.S., Lim S.H.E. (2019). Essential Oils: Partnering with Antibiotics. Essential Oils-Oils of Nature.

[10] Aljaafari M.N., AlAli A.O., Baqais L., Alqubaisy M., AlAli M., Molouki A., Ong-Abdullah J., Abushelaibi A., Lai K.-S., Lim S.-H.E. (2021). An Overview of the Potential Therapeutic Applications of Essential Oils. Molecules.

[11] Máthé A. (2015). Medicinal and Aromatic Plants of the World: Scientific, Production, Commercial and Utilization Aspects.

[12] Yang S.K., Low L.Y., Soo Xï Yap P., Yusoff K., Maï C.W., Laï K.S., Erïn Lïm S.H. (2018). Plant-Derived Antimicrobials: Insights into Mitigation of Antimicrobial Resistance. Rec. Nat. Prod..

[13] Orchard A., van Vuuren S. (2017). Commercial Essential Oils as Potential Antimicrobials to Treat Skin Diseases. Evid.-Based Complement. Altern. Med..

[14] Astani A., Reichling J., Schnitzler P. (2011). Screening for Antiviral Activities of Isolated Compounds from Essential Oils. Evid.-Based Complement. Altern. Med..

[15] Moo C.L., Yang S.K., Osman M.A., Yuswan M.H., Loh J.Y., Lim W.M., Lim S.H.E., Lai K.S. (2020). Antibacterial Activity and Mode of Action of β-caryophyllene on Bacillus cereus. Pol. J. Microbiol..

[16] Yang S.K., Yusoff K., Ajat M., Yap W.S., Lim S.H.E., Lai K.S. (2020). Antimicrobial activity and mode of action of terpene linalyl anthranilate against carbapenemase-producing Klebsiella pneumoniae. J. Pharm. Anal..

[17] Sharmeen J.B., Mahomoodally F.M., Zengin G., Maggi F. (2021). Essential Oils as Natural Sources of Fragrance Compounds for Cosmetics and Cosmeceuticals. Molecules.

[18] Abdellatif F., Boudjella H., Zitouni A., Hassani A. (2014). Chemical composition and antimicrobial activity of the essential oil from leaves of Algerian *Melissa officinalis* L. EXCLI J..

[19] Koyama S., Heinbockel T. (2020). The Effects of Essential Oils and Terpenes in Relation to Their Routes of Intake and Application. Int. J. Mol. Sci..

[20] Patočka J., Kuča K. (2014). Irritant Compounds: Aldehydes. MMSL.

[21] Chang C.T., Chang W.L., Hsu J.C., Shih Y., Chou S.T. (2013). Chemical composition and tyrosinase inhibitory activity of Cinnamomum cassia essential oil. Bot. Stud..

[22] Tajidin N.E., Ahmad S.H., Rosenani A.B., Azimah H., Munirah M. (2012). Chemical composition and citral content in lemongrass (*Cymbopogon citratus*) essential oil at three maturity stages. Afr. J. Biotechnol..

[23] Jalal Z., El Atki Y., Lyoussi B., Abdellaoui A. (2015). Phytochemistry of the essential oil of Melissa officinalis L. growing wild in Morocco: Preventive approach against nosocomial infections. Asian Pac. J. Trop. Biomed..

[24] Mohamed Hanaa A.R., Sallam Y.I., El Leithy A.S., Aly S.E. (2012). Lemongrass (*Cymbopogon citratus*) essential oil as affected by drying methods. Ann. Agric. Sci..

[25] Winkler M. (2018). Carboxylic acid reductase enzymes (CARs). Curr. Opin. Chem. Biol..

[26] Fritz K.S., Petersen D.R. (2013). An overview of the chemistry and biology of reactive aldehydes. Free Radic. Biol. Med..

[27] Niki E., Yoshida Y., Saito Y., Noguchi N. (2005). Lipid peroxidation: Mechanisms, inhibition, and biological effects. Biochem. Biophys. Res. Commun..

[28] Pamplona R., Borras C., Jové M., Pradas I., Ferrer I., Viña J. (2019). Redox lipidomics to better understand brain aging and function. Free Radic. Biol. Med..

[29] Fritz K.S., Petersen D.R. (2011). Exploring the Biology of Lipid Peroxidation-Derived Protein Carbonylation. Chem. Res. Toxicol..

[30] Reed T.T. (2011). Lipid peroxidation and neurodegenerative disease. Free Radic. Biol. Med..

[31] Steven F. (2016). Synthesis of Aldehydes & Ketones. Chemistry LibreTexts. https://chem.libretexts.org/Bookshelves/Organic_Chemistry/Supplemental_Modules_(Organic_Chemistry)/Aldehydes_and_Ketones/Synthesis_of_Aldehydes_and_Ketones/Synthesis_of_Aldehydes_and_Ketones.

[32] Dhifi W., Bellili S., Jazi S., Bahloul N., Mnif W. (2016). Essential Oils’ Chemical Characterization and Investigation of Some Biological Activities: A Critical Review. Medicines.

[33] Chen Y.F., Wang Y.W., Huang W.S., Lee M.M., Wood W.G., Leung Y.M., Tsai H.Y. (2016). Trans-Cinnamaldehyde, An Essential Oil in Cinnamon Powder, Ameliorates Cerebral Ischemia-Induced Brain Injury via Inhibition of Neuroinflammation Through Attenuation of iNOS, COX-2 Expression and NFκ-B Signaling Pathway. NeuroMolecular Med..

[34] Ooi L.S., Li Y., Kam S.L., Wang H., Wong E.Y., Ooi V.E. (2006). Antimicrobial Activities of Cinnamon Oil and Cinnamaldehyde from the Chinese Medicinal Herb Cinnamomum cassia Blume. Am. J. Chin. Med..

[35] Mukarram M., Khan M.M.A., Zehra A., Choudhary S., Aftab T., Naeem M. (2020). Biosynthesis of Lemongrass Essential Oil and the Underlying Mechanism for Its Insecticidal Activity. Medicinal and Aromatic Plants.

[36] Abdulazeez M.A., Abdullahi A.S., James B.D., Preedy V.R. (2016). Chapter 58-Lemongrass (*Cymbopogon* spp.) Oils. Essential Oils in Food Preservation, Flavor and Safety.

[37] Majewska E., Kozlowska M., Gruszczynska Sekowska E., Kowalska D., Tarnowska K. (2019). Lemongrass (*Cymbopogon citratus*) essential oil: Extraction, composition, bioactivity and uses for food preservation—A review. Pol. J. Food Nutr. Sci..

[38] Abdollah P., Somayeh S., Lyle C. (2016). Effect of drying methods on qualitative and quantitative properties of essential oil from the aerial parts of coriander. Elsevier Enhanced Reader. https://reader.elsevier.com/reader/sd/pii/S2214786116300341?token=EC17BC5E9F2C810DF1B04EDD9D083DEB4A69385F564396FB123B1D5D3017C730EE17C6651979A103BAC207C9A7BCB91F&originRegion=us-east-1&originCreation=20210616123940.

[39] Hasanein P., Riahi H. (2015). Antinociceptive and Antihyperglycemic Effects of Melissa officinalis Essential Oil in an Experimental Model of Diabetes. Med. Princ. Pract..

[40] Pourghanbari G., Nili H., Moattari A., Mohammadi A., Iraji A. (2016). Antiviral activity of the oseltamivir and Melissa officinalis L. essential oil against avian influenza A virus (H9N2). VirusDisease.

[41] Chang W.L., Cheng F.C., Wang S.P., Chou S.T., Shih Y. (2017). Cinnamomum cassia essential oil and its major constituent cinnamaldehyde induced cell cycle arrest and apoptosis in human oral squamous cell carcinoma HSC-3 cells. Environ. Toxicol..

[42] Doyle A.A., Stephens J.C. (2019). A review of cinnamaldehyde and its derivatives as antibacterial agents. Fitoterapia.

[43] Valdivieso Ugarte M., Plaza Diaz J., Gomez Llorente C., Lucas Gómez E., Sabés Alsina M., Gil Á. (2021). In vitro examination of antibacterial and immunomodulatory activities of cinnamon, white thyme, and clove essential oils. J. Funct. Foods.

[44] El Khouly A.S., Kenawy E., Safaan A.A., Takahashi Y., Hafiz Y.A., Sonomoto K., Zendo T. (2011). Synthesis, characterization and antimicrobial activity of modified cellulose-graft-polyacrylonitrile with some aromatic aldehyde derivatives. Carbohydr. Polym..

[45] Yap P.S.X., Yang S.K., Lai K.S., ErinLim S.H. (2017). Essential Oils: The Ultimate Solution to Antimicrobial Resistance in *Escherichia coli*?. Recent Advances on Physiology, Pathogenesis and Biotechnological Application.

[46] Chuesiang P., Siripatrawan U., Sanguandeekul R., Yang J.S., McClements D.J., McLandsborough L. (2019). Antimicrobial activity and chemical stability of cinnamon oil in oil-in-water nanoemulsions fabricated using the phase inversion temperature method. LWT.

[47] Rao J., McClements D.J. (2010). Stabilization of Phase Inversion Temperature Nanoemulsions by Surfactant Displacement. J. Agric. Food Chem..

[48] Mahizan N.A., Yang S.K., Moo C.L., Song A.A.L., Chong C.M., Chong C.W., Abushelaibi A., Lim S.H.E., Lai K.S. (2019). Terpene Derivatives as a Potential Agent against Antimicrobial Resistance (AMR) Pathogens. Molecules.

[49] Netopilova M., Houdkova M., Urbanova K., Rondevaldova J. (2020). In vitro antimicrobial combinatory effect of Cinnamomum cassia essential oil with 8-hydroxyquinoline against *Staphylococcus aureus* in liquid and vapour phase. J. Appl. Microbiol..

[50] Firmino D.F., Cavalcante T.T.A., Gomes G.A., Firmino N.C.S., Rosa L.D., de Carvalho M.G., Catunda F.E.A. (2018). Antibacterial and Antibiofilm Activities of Cinnamomum Sp. Essential Oil and Cinnamaldehyde: Antimicrobial Activities. Sci. World J..

[51] Macwan S., Dabhi B., Aparnathi K., Prajapati J. (2016). Essential Oils of Herbs and Spices: Their Antimicrobial Activity and Application in Preservation of Food. Int. J. Curr. Microbiol. Appl. Sci..

[52] Gabriel K.T., Kartforosh L., Crow S.A., Cornelison C.T. (2018). Antimicrobial Activity of Essential Oils Against the Fungal Pathogens Ascosphaera apis and Pseudogymnoascus destructans. Mycopathologia.

[53] Filomena S., Fernanda D. (2017). Antimicrobial activity of coriander oil and its effectiveness as food preservative: *Crit*. Rev. Food Sci. Nutr..

[54] Behbahani B.A., Shahidi F. (2019). [PDF] Melissa officinalis Essential Oil: Chemical Compositions, Antioxidant Potential, Total Phenolic Content and Antimicrobial Activity. Semantic Scholar. https://www.semanticscholar.org/paper/Melissa-officinalis-Essential-Oil%3A-Chemical-Total-Behbahani-Shahidi/f516785cc9809744d4cd7e131593d7040bd5f3db?p2df.

[55] Kim M.E., Na J.Y., Lee J.S. (2018). Anti-inflammatory effects of trans-cinnamaldehyde on lipopolysaccharide-stimulated macrophage activation via MAPKs pathway regulation. Immunopharmacol. Immunotoxicol..

[56] Lang M., Ferron P.J., Bursztyka J., Montjarret A., Duteil E., Bazire A., Bedoux G. (2019). Evaluation of immunomodulatory activities of essential oils by high content analysis. J. Biotechnol..

[57] Valdivieso Ugarte M., Gomez Llorente C., Plaza Díaz J., Gil Á. (2019). Antimicrobial, Antioxidant, and Immunomodulatory Properties of Essential Oils: A Systematic Review. Nutrients.

[58] Shen C., Christensen L.G., Bak S.Y., Christensen N., Kragh K. (2020). Immunomodulatory effects of thymol and cinnamaldehyde in chicken cell lines. J. Appl. Anim. Nutr..

[59] Yang L., Wu Q.Q., Liu Y., Hu Z.F., Bian Z.Y., Tang Q.Z. (2015). Cinnamaldehyde attenuates pressure overload-induced cardiac hypertrophy. Int. J. Clin. Exp. Pathol..

[60] Campbell S., Soman Faulkner K. (2021). Antiparasitic Drugs. StatPearls.

[61] Saeidnia S., Gohari A.R. (2012). Chapter 6—Trypanocidal Monoterpenes: Lead Compounds to Design Future Trypanocidal Drugs. Studies in Natural Products Chemistry.

[62] Farhath S., Vijaya P., Vimal M. (2013). Immunomodulatory activity of geranial, geranial acetate, gingerol, and eugenol essential oils: Evidence for humoral and cell-mediated responses. Avicenna J. Phytomed..

[63] LoPachin R.M., Gavin T. (2014). Molecular Mechanisms of Aldehyde Toxicity: A Chemical Perspective. Chem. Res. Toxicol..

[64] National Toxicology Program: 14th Report on Carcinogens. National Toxicology Program (NTP). https://ntp.niehs.nih.gov/go/roc14.

[65] Tamura M., Ito H., Matsui H., Hyodo I. (2014). Acetaldehyde is an oxidative stressor for gastric epithelial cells. J. Clin. Biochem. Nutr..

[66] (2014). Formaldehyde. American Cancer Society. https://www.cancer.org/cancer/cancer-causes/formaldehyde.html.

[67] Bernardini L., Barbosa E., Charão M.F., Brucker N. (2020). Formaldehyde toxicity reports from in vitro and in vivo studies: A review and updated data. Drug Chem. Toxicol..

[68] (2011). Formaldehyde and Cancer Risk—National Cancer Institute. https://www.cancer.gov/about-cancer/causes-prevention/risk/substances/formaldehyde/formaldehyde-fact-sheet.

[69] Duong A., Steinmaus C., McHale C.M., Vaughan C.P., Zhang L. (2011). Reproductive and developmental toxicity of formaldehyde: A systematic review. Mutat. Res..

[70] Andersen A. (2006). Final report on the safety assessment of benzaldehyde. Int. J. Toxicol..

[71] Kluwe W.M., Montgomery C.A., Giles H.D., Prejean J.D. (1983). Encephalopathy in rats and nephropathy in rats and mice after subchronic oral exposure to benzaldehyde. Food Chem. Toxicol. Int. J. Publ. Br. Ind. Biol. Res. Assoc..

[72] Laham S., Broxup B., Robinet M., Potvin M., Schrader K. (1991). Subacute inhalation toxicity of benzaldehyde in the Sprague-Dawley rat. Am. Ind. Hyg. Assoc. J..

[73] Kodak E. (1991). Letter from Eastman Kodak Company to USEPA Submitting Enclosed Toxicity & Health Hazard Summary and Toxicity Report on Bis(2-Methoxyethyl)ether with Attachments.

[74] Letizia C.S., Cocchiara J., Wellington G.A., Funk C., Api A.M. (2000). Food and chemical toxicology. Food Chem. Toxicol. Int. J. Publ. Br. Ind. Biol. Res. Assoc..

[75] Qu S., Yang K., Chen L., Liu M., Geng Q., He X., Li Y., Liu Y., Tian J. (2019). Cinnamaldehyde, a Promising Natural Preservative Against Aspergillus flavus. Front. Microbiol..

[76] Zhu R., Liu H., Liu C., Wang L., Ma R., Chen B., Li L., Niu J., Fu M., Zhang D. (2017). Cinnamaldehyde in diabetes: A review of pharmacology, pharmacokinetics and safety. Pharmacol. Res..

[77] Liao J.-C., Deng J.-S., Chiu C.-S., Hou W.-C., Huang S.-S., Shie P.-H., Huang G.-J. (2012). Anti-Inflammatory Activities of Cinnamomum cassia Constituents In Vitro and In Vivo. Evid.-Based Complement. Altern. Med..

[78] Chen L., Wang Z., Liu L., Qu S., Mao Y., Peng X., Li Y., Tian J. (2019). Cinnamaldehyde inhibits Candida albicans growth by causing apoptosis and its treatment on vulvovaginal candidiasis and oropharyngeal candidiasis. Appl. Microbiol. Biotechnol..

[79] Dorri M., Hashemitabar S., Hosseinzadeh H. (2018). Cinnamon (*Cinnamomum zeylanicum*) as an antidote or a protective agent against natural or chemical toxicities: A review. Drug Chem. Toxicol..

[80] Wang L., Jin J., Liu X., Wang Y., Liu Y., Zhao Y., Xing F. (2018). Effect of Cinnamaldehyde on Morphological Alterations of Aspergillus ochraceus and Expression of Key Genes Involved in Ochratoxin A Biosynthesis. Toxins.

